# Perceived Fatality Prior to COVID-19 Infection in 13 Latin American Countries (FAT-LAT-COVID-19): Revalidation of a Shortened Scale

**DOI:** 10.3389/fpsyt.2021.724061

**Published:** 2022-02-21

**Authors:** Christian R. Mejia, Telmo Raúl Aveiro-Róbalo, Luciana D. Garlisi-Torales, Renzo Felipe Carranza Esteban, Oscar Mamani-Benito, Martín A. Vilela-Estrada, Víctor Serna-Alarcón, Damary S. Jaramillo-Aguilar, Javiera L. Rojas-Roa

**Affiliations:** ^1^Center for Translational Medicine Research, Norbert Wiener University, Lima, Peru; ^2^Universidad del Pacifico, Asunción, Paraguay; ^3^Federación Latinoamericana de Sociedades Científicas de Estudiantes de Medicina, Universidad del Pacifico, Asunción, Paraguay; ^4^Grupo de Investigación Avances en Investigación Psicológica, Facultad de Humanidades, Universidad San Ignacio de Loyola, Lima, Peru; ^5^Facultad de Ciencias de la Salud, Universidad Peruana Unión, Juliaca, Peru; ^6^Escuela de Medicina Humana, Universidad Privada Antenor Orrego, Trujillo, Peru; ^7^Escuela de Medicina, Facultad de Ciencias Médicas, Universidad de Cuenca, Cuenca, Ecuador; ^8^Universidad Mayor de Temuco, Temuco, Chile

**Keywords:** coronavirus infections, pandemics, fatalistic perception, validation study, Latin America

## Abstract

**Introduction:**

COVID-19 has generated great repercussions for the population globally; millions of deaths have been reported worldwide. The idea of death is especially exacerbated when there are close to death experiences that remind us how close we are to fatality. This is why it is important to measure fatalistic ideas of those who have not yet been infected.

**Objective:**

To revalidate a scale that measures fatalistic perception prior to COVID-19 infection in a population of 13 Latin American countries.

**Methodology:**

We conducted an instrumental study. We used a previously validated scale in Peru, with seven items divided into two factors and with five possible Likert-type responses (from strongly disagree to strongly agree). It was administered to a large population in 13 Spanish-speaking countries in Latin America; for each of the seven questions, 886 people were surveyed. With these results, descriptive and analytical statistics were performed.

**Results:**

The mean, standard deviation, skewness, and kurtosis of the seven initial questions were adequate in most cases. In the confirmatory factor analysis, the lack of fit was improved with the indexes' modification technique, which let us delete items 1 and 6. Thus, we could obtain satisfactory goodness-of-fit indices (CFI = 0.972, TLI = 0.931, GFI = 0.990, AGFI = 0.961, RMSEA = 0.080, and RMR = 0.047). Therefore, the final two-factor structure had a fairly adequate Cronbach's α (0.72, with a 95% confidence interval = 0.70–0.73).

**Conclusions:**

The scale that measures fatalism of Latin American countries in the face of the pandemic generated by COVID-19 was revalidated and shortened.

## Introduction

The idea of death is one of the most terrifying conceptions for human beings (1). It is especially exacerbated when there are close to death experiences in regard to a relative, a friend, or any other person, which reminds us how close we are to fatality (2). Fatalism is believing that something is going to happen, which is an inescapable event. This is really important in this context, since this pandemic caused by COVID-19 has generated, in half of 2020, almost half a million deaths, reported worldwide (3). It has shown the whole world not only the fragility of many health systems (4), but also how death can be around, as we are facing this every day.

It is very important to measure this fatalism in different populations, since this affected countries differently (5), both because of the actions of their governments from the first days (6) and because of the response that people had to these norms (7), including other influencing social determinants (8). Therefore, each population or context had to perceive this disease in different ways; hence, it is necessary to generate tools that can allow us to show how the various countries of Latin America felt regarding the possibility of a fatal outcome due to coronavirus contagion (9). That is why the objective of the research was to revalidate a scale that measures fatalistic perception prior to COVID-19 infection in the population of 13 Latin American countries.

## Methods

### Design

An instrumental and cross-sectional study was carried out (10). The population consisted of a large group of individuals in various realities of Latin America. The study subjects were contacts of medical students who belonged to the Latin American Federation of Scientific Societies of Medical Students, who also participated in the research. After being contacted, they contacted others within their social circles and, then, this operation was repeated multiple times. Subjects who resided in a Latin American country, who speak Spanish, who have stated that they had not yet been infected due to COVID-19, and who agreed to participate in the research were included. A total of 674 respondents were excluded, because they did not answer the seven initial questions of the fatalism test. A non-probability convenience sampling was used. It was required that we have a minimum of 15–20 respondents for each question; however, we obtained the answers of 886.6 people for each of the questions, which means that the minimum sample size was exceeded. The survey was administered to 6,206 people from Bolivia, Chile, Colombia, Costa Rica, Ecuador, El Salvador, Guatemala, Honduras, Mexico, Panama, Paraguay, Peru, and Venezuela.

### Initial Instrument to Revalidate

The COVID-19 fatalism scale (F-COVID-19) was validated in a large Peruvian population (9). The surveys were administered from June 7 to June 17, after which a data quality control was carried out, where the exclusion criteria were taken into account. Of the recruited countries, the one that contributed the most surveys was Peru (3,976), followed by Chile (633), Paraguay (622), Mexico (403), Bolivia (356), Ecuador (282), Panama (136), Costa Rica (124), and El Salvador (123). The other countries contributed fewer than 100 surveys. In Peru, there were a greater number of surveys due to the fact that it is the main affected country in the region (after Brazil, which was not included because a different language is spoken there). The majority of respondents were women (60.4%), with a median age of 21 years (interquartile range: 19–28 years). The population was eminently urban, residing in large cities where there was a possibility that it had already had cases of COVID-19. The instrument is Likert type and is made up of seven items, which were elaborated by a group of researchers in the coastal, highland, and jungle regions of Peru. It was evaluated by multiple experts, who expressed their agreement with the relevance, representativeness, and clarity of each of the initial items. This is why this process did not have to be repeated in this validation study. It should be noted that each of the questions had five response options (strongly disagree, disagree, indifferent, agree, and strongly agree).

### Revalidation Procedure

The project of this research was approved by the Ethics Committee of the Antenor Orrego Private University (Bioethics Committee Resolution Number 0237-2020-UPAO). It is important to emphasize that, at all times, the anonymity and free participation of the respondents were respected. After the approval, the questions of the scale, previously validated in Peru (F-COVID-19), were used; they were analyzed and reviewed by the research team. Then, a pilot test was applied in each of the realities to see if the questions and alternatives were fully understood. Subsequently, the instrument was administered on a massive scale, through a collection process that was led by the representatives of the Latin American Federation of Scientific Societies of Medical Students. This institution was the one that co-led this process of investigation. It is important to mention that data collection was carried out through the electronic form of Google Forms and was directed to our study population, mainly due to the fact that in many countries there were restrictions on mobility. It should be mentioned that the Spanish version of this instrument was used to carry out the surveys in the Latin American countries; however, the items were subjected to a back-translation in order to be published in an English-language journal; the Spanish version can be consulted in the supplementary material.

### Data Analysis

FACTOR Analysis version 10.1, a statistical program, was used to analyze the mean, standard deviation, asymmetry, and kurtosis of the seven items of the scale. The Confirmatory Factor Analysis (CFA) was carried out with the statistical software AMOS version 21, to evaluate the goodness of fit of the original model, and we used structural equation modeling (SEM) as well. The absolute and incremental goodness of fit was determined through the comparative fit index (CFI), Tucker–Lewis Index (TLI), goodness-of-fit index (GFI), and adjusted goodness-of-fit index (AGFI). Likewise, the parameters for the mean square error of approximation (RMSEA) and the mean square error index (RMR) were used. The recommendations of Jimenez et al. (11) were taken into account, as they argued that the value of the CFI, TLI, GFI, and AGFI should be greater than 0.90 and the RMSEA ≤ 0.08 to have an acceptable model fit. Finally, the SPSS software version 23.0 was used to estimate the reliability of the scale through Cronbach's α coefficient and their respective confidence intervals (12).

## Results

Table 1 shows the mean, standard deviation, skewness, and kurtosis for the seven evaluated items, which came from the initial scale. We can observe that item 2 has the highest average score (*M* = 2.59) and item 6 has the lowest (*M* = 0.64). Regarding variability, item 1 (SD = 1.46) shows the highest dispersion. The asymmetry and kurtosis of items 1, 2, 3, 4, 5, and 7 of the FAT-LAT-COVID-19 Scale are adequate; however, the values of item 6 exceed the range> ± 1.5 (13).

**Table 1:** Preliminary analysis of the items of the FAT-LAT-COVID-19 scale.

**Items**	**Mean**	**SD**	**Asymmetry**	**Kurtosis**
Item 1	2.126	1.465	−0.287	−1.390
Item 2	2.592	1.300	−0.820	−0.537
Item 3	1.968	1.286	−0.024	−1.222
Item 4	1.900	1.297	0.010	−1.180
Item 5	1.554	1.222	0.391	−0.881
Item 6	0.641	0.954	1.611	2.178
Item 7	0.934	1.153	1.068	0.163

*SD, Standard deviation*.

### Confirmatory Factor Analysis

**Figure 1:**
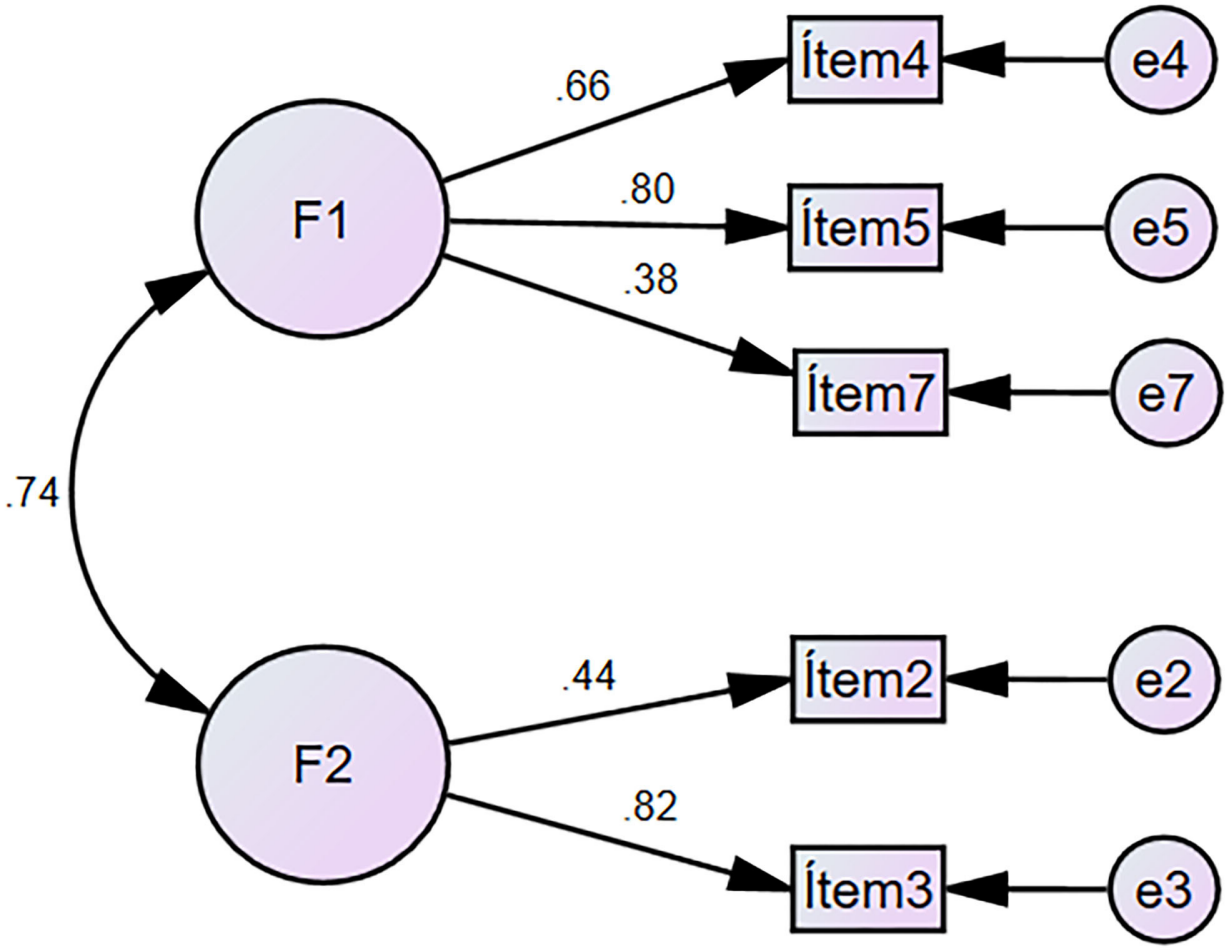
Model 1 of the FAT-LAT-COVID-19 Scale.

Using the CFA, we sought to observe to what extent the original model of the FAT-LAT-COVID-19 scale (7 items distributed in three factors) adjusted to the collected data. However, the results of the original model showed that the goodness of fit for the first model was poor (Table 1). Therefore, the lack of fit was analyzed with the index modification technique where it was found that items 1 and 6 were associated; hence, they were eliminated and satisfactory goodness-of-fit indices were obtained (CFI = 0.972, TLI = 0.931, GFI = 0.990, AGFI = 0.961, RMSEA = 0.080 and RMR = 0.047). To sum up, model 1 met the goodness-of-fit criteria, had goodness-of-fit indices (Table 2), and had five items distributed in a two-factor structure (Figure 1). Additionally, the correlations between factors were significant (*p* < 0.05).

**Table 2:** Fit indices of the factor models of the FAT-LAT-COVID-19 scale.

**Model**	**χ^2^**	**gl**	***p***	**CFI**	**TLI**	**GFI**	**AGFI**	**RMSEA**	**CMIN/DF**	**RMR**
Original	1,791.287	13	<0.001	0.813	0.699	0.813	0.831	0.148	137.791	0.118
1	164.590	4	<0.001	0.972	0.931	0.990	0.961	0.080	41.147	0.047

Finally, the reliability of the construct was calculated using Cronbach's α coefficient; thus, we could obtain an acceptable value (α = 0.72, 95% CI = 0.70–0.73). Therefore, the final scale is shown in Table 3.

**Table 3:** Final FAT-LAT-COVID-19, shortened version.

**Factor**	**IF YOU GOTCORONAVIRUS …**	**Strongly agree**	**Agree**	**Indifferent**	**Disagree**	**Strongly disagree**
1st	I believe that if I get sick, I will infect my family/friends.					
1st	I think I will be admitted to a hospital for a complication.					
2nd	I think that getting this virus will make me depressed.					
2nd	I think that, by catching this virus, I could die.					
2nd	I think this shows that the “end of the world” is near.					

## Discussion

Measuring fatalistic perception is of utmost importance, because it allows the quantification of a belief about the possible outcome of suffering from the disease, which is often inadequate. This could be influenced by multiple factors, such as having comorbidities, having listened to too much negative information from the media (14), people's religious beliefs (15), or even social class (16).

The first factor of the test was reduced to one question. Now, there is no longer the possibility of contagion at work, leaving the question about the fact that someone can get infected and infect family or friends as the first question. The probability of the spread of the virus is very high due to community dissemination through family gatherings, social events, and holidays (17, 18). This must be measured in each reality, since it is known that COVID-19 has affected different realities, so this question will also be influenced by the number of people who are infected in a community or environment.

The second question of the first factor refers to a possible complication of the disease, as we know that ~20% of the population will have complications. Hence, it is important to measure this with questions that seek to see if the population who has fatalistic ideas has a real risk of complication, since, if the risk does not exist, there could exist a possible exaggeration of the risk. Various complications have been reported, such as neurological and cardiovascular as the main ones (19–22). Therefore, if people have a high perception in regard to this question, despite not having comorbidities then, it would be appropriate to measure the degree of anxiety they have, since they could be having thoughts that are not relevant to their risk level of complication. It would be interesting, for future research, to consider instruments that can promptly assess anxiety during pandemics or during this particular pandemic.

The first question of the second factor addresses the mental sphere, where depression is a very important factor in this regard as it has been researched by many previous studies, which showed that depression is one of the most common mental pathologies in this pandemic. Therefore, its surveillance is of utmost importance, since numerous studies have reported high rates of mental health impairment in different populations, such as what has been reported in health personnel (23), young adults (24), and university students (25–27), among others. Thus, if this question is responded to positively, it is recommended that depressive disorders, or even depressive or suicidal thoughts, should be measured.

The penultimate question of the test shows the possibility of death as an important problem. This is crucial as we have news about deaths every day in various parts of the world, which exposes the most important characteristic of the virus: its high risk of contagion rather than mortality risk. This must also be measured, since the fear of the disease itself can be even more dangerous than suffering from COVID as it has even led to suicide in some extreme cases. In India, the case of a man diagnosed with a viral pathology, although not confirmed COVID, was so disturbed that, to protect his family, he quarantined himself and threw stones at his family and friends when they tried to get close to him. Later, due to his fear and panic of having acquired COVID, he ended his life by hanging himself from a tree (28). By exemplifying with this extreme case, we want to show that some people could have extreme thoughts or take extreme measures through their actions, thinking that they will protect their loved ones with these actions. However, this instrument should be administered to try to detect extreme fatalistic thoughts in cases like these.

Finally, a question that persisted from the original test is the one that refers to a fatalistic possibility in the religious sense, which may be due to the fact that in Latin America, there are still large population groups that follow various religions such as Catholicism, Protestantism, Evangelicalism, and non-Christian groups (29), among many others. Therefore, we recommended that this should also be measured with a question about religious beliefs, so that some cases that have a “magical-religious” or extremist thinking can be detected (30). These situations have been seen before, and they were thought to be indicative of the end of the world or the apocalypse, such as the event of the end of 2012 (31).

The main limitation of the study was being unable to reach all the Latin American countries and their sub-populations, in addition to the heterogeneous numbers for each country. However, the large number of respondents for each question and adequate overall adjustment of the items, factors, and survey allowed us to estimate that the validated scale is quite adequate for its objective. The administration of this validated scale is recommended as it can be applied in large populations, combined with other instruments that evaluate different aspects of mental health. Furthermore, it was not possible to be certain whether the respondents were asymptomatic at the time of the administration of the survey due to the type of design (which did not allow follow-up). Some final values did not have an adequate Cronbach's α; however, we decided to keep the items in the final instrument because the global value of Cronbach's α remained between 0.70 and 0.90. This indicated that, even though we had values out of range, the global one worked properly.

Taking into account all that was reported, we concluded that the validation of the fatalistic perception test was optimally carried out in the face of the possible contagion of COVID-19 in a large population of Latin America. In addition, we can mention that it can help to quickly and efficiently measure this issue in different Spanish-speaking populations. We suggest the use of the scale to have a perspective of fatalistic perception during this pandemic.

## Data Availability Statement

The raw data supporting the conclusions of this article will be made available by the authors, without undue reservation.

## Ethics Statement

The studies involving human participants were reviewed and approved by Comité de Bioética en Investigación of the Universidad Privada Antenor Orrego. Written informed consent for participation was not required for this study in accordance with the national legislation and the institutional requirements.

## Author Contributions

CM, RC, and OM-B: conceptualization, methodology, formal analysis, investigation, data curation, writing—original draft, and writing—review and editing. TA-R, LG-T, MV-E, VS-A, DJ-A, and JR-R: formal analysis, resources, investigation, data curation, writing—original draft, and writing—review and editing. All authors contributed to the article and approved the submitted version.

## Conflict of Interest

The authors declare that the research was conducted in the absence of any commercial or financial relationships that could be construed as a potential conflict of interest.

## Publisher's Note

All claims expressed in this article are solely those of the authors and do not necessarily represent those of their affiliated organizations, or those of the publisher, the editors and the reviewers. Any product that may be evaluated in this article, or claim that may be made by its manufacturer, is not guaranteed or endorsed by the publisher.
